# Effect of Hand and Foot Massage Therapy on Psychological Factors and EEG Activity in Elderly People Requiring Long-Term Care: A Randomized Cross-Over Study

**DOI:** 10.3390/brainsci9030054

**Published:** 2019-03-04

**Authors:** Hideki Nakano, Takayuki Kodama, Tomohiro Ueda, Ikuko Mori, Tomiko Tani, Shin Murata

**Affiliations:** 1 Department of Physical Therapy, Faculty of Health Science, Kyoto Tachibana University, Kyoto 607-8175, Japan; kodama-t@tachibana-u.ac.jp (T.K.); murata-s@tachibana-u.ac.jp (S.M.); 2 Graduate School of Health Science, Kyoto Tachibana University, Kyoto 607-8175, Japan; ueda_tomo1212@yahoo.co.jp; 3 Naris Cosmetics Company Limited, Osaka 553-0001, Japan; ikuko_mori@naris.co.jp (I.M.); t_tani@naris.co.jp (T.T.); 4 Japan Wellness Therapist Association, Osaka 553-0001, Japan

**Keywords:** massage therapy, elderly people, emotion, mood, EEG

## Abstract

Massage therapy is widely used as a complementary therapy in the elderly. Here, we investigate the effect of hand and foot massage therapy on psychological factors and electroencephalographic (EEG) activity in elderly people requiring long-term care. We included 12 elderly people requiring long-term care, who were randomly divided into two groups (A and B). Group A received hand massage and group B received foot massage, both for 15 min each. After 1 week, group A received foot massage and group B received hand massage, both for 15 min each. We assessed emotions and mood states with a Likert scale after each massage and resting-state EEG activity was measured before and after each massage. Our results showed that both hand and foot massage led to a high degree of pleasant, relaxed, and refreshed feelings. Moreover, resting-state alpha activity significantly increased in the left insular cortex after hand massage (*p* < 0.05), and in the right and left posterior cingulate cortex after foot massage (*p* < 0.05). This study suggests that hand and foot massage therapy modulate psychological factors and EEG activity in elderly people requiring long-term care.

## 1. Introduction

Massage therapy is a widely-used complementary and alternative therapy for elderly people [1,2,3]. The American Massage Therapy Association defines massage as “manual soft tissue manipulation, including holding, causing movement, and/or applying pressure to the body” [4]. It is reported that massage therapy effectively improves the health and well-being of elderly people, which may be important as the elderly population increases in various countries worldwide [5].

In a study examining the effect of massage therapy on the elderly, Sharpe et al. [6] demonstrated that massage therapy enhances well-being and reduces stress perception among elderly people. Moreover, Munk et al. [7] reported a relationship between massage therapy and health outcomes in the elderly. Similarly, Sefton et al. [8,9] have reported that massage therapy improves postural balance and blood pressure in elderly people.

Consistent with these positive effects of massage, research investigating the physiological effects of massage therapy with electroencephalography (EEG) has suggested that massage therapy reduces anxiety, increases frontal delta activity, and decreases frontal alpha and beta activity [10]. Jones et al. [11] also reported that massage therapy shifts frontal alpha asymmetry from right-hemisphere dominance to left-hemisphere dominance. Moreover, Diego et al. [12] confirmed these findings, suggesting that massage therapy reduces anxiety and stress, increases frontal delta activity, decreases frontal alpha and beta activity, and shifts frontal alpha asymmetry from right-hemisphere dominance to left-hemisphere dominance. Similarly, our previous study demonstrated that manual massage increases resting-state alpha activity in the left anterior cingulate cortex compared with machine massage [13]. Together, these findings provide evidence that massage therapy effectively improves the health and well-being of the elderly, and that massage therapy acts to modulate EEG activity.

However, previous studies have investigated massage therapy on various body parts including the hand [14,15,16,17,18,19,20], foot [21,22], and lower back [15,23,24]. The different effects of massage therapy on each body part have, therefore, not been sufficiently clarified. Hence, this study aimed to investigate the effect of hand and foot massage therapy on psychological factors and EEG activity in elderly people requiring long-term care.

## 2. Materials and Methods

### 2.1. Participants

The population from which the study sample was drawn included 69 elderly people who attended a day service center. We included participants aged 65 years and older, and those with Japanese long-term care insurance (LTCI) services [25]. Elderly people using LTCI services had physical or mental impairment and needed assistance with activities of daily living [26]. We excluded participants with Mini-Mental State Examination (MMSE) scores below 24, and those with orthopedic, neurological, cardiovascular, or psychiatric diseases that might influence the results. Finally, 12 elderly people (female, *n* = 9; male, *n* = 3; mean age ± standard deviation: 81.9 ± 3.9 years) were recruited to the study. Recruitment of participants was conducted by a third party, and all those who were approached to participate consented to taking part. The power calculation was performed using G*Power [27,28].

The study was conducted according to the principles of the Declaration of Helsinki and was approved by the local institutional ethics committee of Kyoto Tachibana University. All participants provided informed written consent and were free to withdraw from the study at any time.

### 2.2. Study Protocol

This study was a randomized cross-over study. Initially, participants were randomly divided into two groups (A and B) using random numbers generated by Microsoft Excel 2010 (Microsoft, Redmond, WA, USA). Group A received hand massage and group B received foot massage, both for 15 min each. After 1 week, the body part for massage was changed such that group A received foot massage and group B received hand massage, both for 15 min each.

Participants received hand massage while sitting on a chair with a back and armrests. Foot massage was received while in a supine position on a bed. Each massage was performed by two therapists who had worked as instructors at the Japan Wellness Therapist Association for five years. One therapist (female, 65 years old) performed only hand massage and the other (female, 60 years old) performed only foot massage. Fragrance-free massage oil (2E32000, Naris Cosmetics Co., Ltd., Osaka, Japan) was used during each massage to prevent friction and discomfort.

A standard massage technique [13], without pressure on points indicated by reflexology [29], was used in this study. Hand massage was performed according to the following procedure. First, the therapist stroked the entire lower arm, from the wrist to the elbow, using her entire hand (5 min). Next, she stroked the entire hand from the fingers to the wrist (5 min). Finally, she stroked the fingers one by one from the base of the finger toward the tip (5 min).

Foot massage was performed according to the following procedure. First, the therapist stroked the entire lower leg, from the ankle to the knee, using her entire hand (5 min). Next, she stroked the entire foot, from the toes to the ankle (5 min). Finally, she stroked the toes, one by one, from the base of the toe toward the tip (5 min). The therapists were requested to keep the pressure intensity and rate of massage as consistent as possible for all participants. Each massage was conducted for 15 min. Emotions and mood states were assessed after each massage (Post1 and Post2), and resting-state EEG activity was measured before (Pre1 and Pre2) and after (Post1 and Post2) each massage. The measurement of emotions and mood states and resting-state EEG activity was conducted by the study investigators.

### 2.3. Measures

To assess emotions and mood states, a Likert scale [30] was used to measure the degree to which participants felt pleasant, relaxed, and refreshed after each massage [13]. The Likert scale consisted of a 5-point scale ranging from 1 (strongly disagree) to 5 (strongly agree), with higher scores indicating positive emotions and mood states.

Resting-state EEG activity before and after each massage was measured for 90 s in the eyes open condition and while the patients were seated in a chair with a backrest. The EEG was obtained with an electroencephalograph (EEG-9100, Nihon Kohden Co., Ltd., Tokyo, Japan) and an active dry electrode system (Miyuki Giken Co., Ltd., Tokyo, Japan). The EEG was recorded with 19 channels (Fp1, Fp2, F7, F3, Fz, F4, F8, T3, C3, Cz, C4, T4, T5, P3, Pz, P4, T6, O1, and O2) based on the international 10–20 system and at a sampling rate of 1000 Hz. Reference electrodes were attached to both earlobes. Participants in both groups were seated for several minutes after massage before EEG recording.

Recorded EEG data were processed in EEGLAB as follows: down sampling to 256 Hz, band pass filter set to 1–40 Hz, independent component analysis to remove artifacts, and re-referencing to the average reference [31]. Next, exact low-resolution brain electromagnetic tomography (eLORETA) was used to analyze the cortical distribution of current source density [32]. In eLORETA, the solution space consists of 6239 cortical gray matter voxels at 5 mm spatial resolution, in a realistic head model [33], using the Montreal Neurological Institute (MNI) 152 template [34]. The eLORETA image was calculated in the alpha frequency band (8–13 Hz) because the resting alpha activity reflects the emotions and mood states [35].

### 2.4. Statistical Analysis

The baseline characteristics of the groups A and B were compared to check if the two groups were comparable. The Kolmogorov–Smirnov Test was used to test the normality of distributions, and differences between groups were analyzed using Student’s *t*-tests for normally distributed variables and the Mann–Whitney U test for variables that were not normally distributed. The degree to which participants felt pleasant, relaxed, and refreshed after each massage was analyzed with the Wilcoxon signed-rank test. Statistical analyses were performed with SPSS ver. 24.0 (IBM, Chicago, IL, USA). The pre- and post-massage cortical distributions of current source density were compared in the alpha frequency band with voxel-by-voxel dependent sample F-ratio tests, based on the eLORETA log-transformed current density power. In the resulting statistical three-dimensional images, cortical voxels exhibiting significant differences were identified with a non-parametric approach (statistical non-parametric mapping; SnPM). The level of significance was set at *p* < 0.05.

## 3. Results

**Figure 1:**
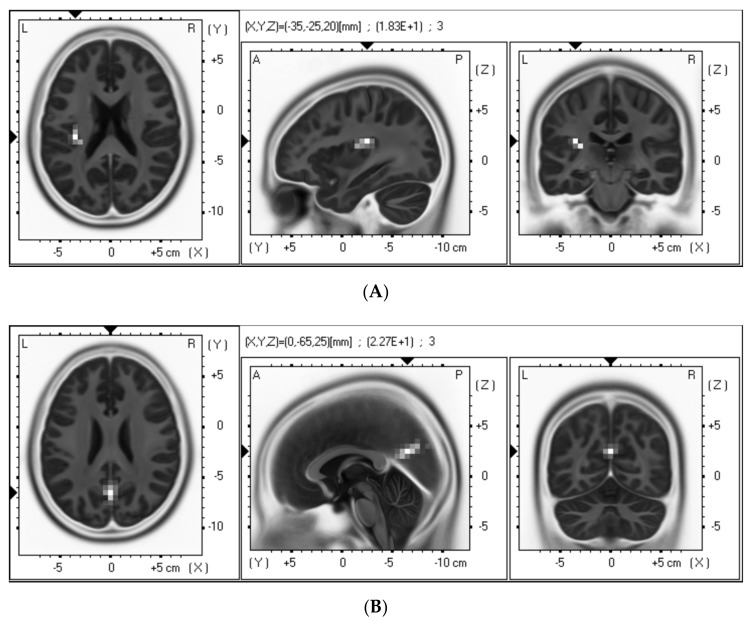
Statistical non-parametric maps (SnPMs) of exact low-resolution brain electromagnetic tomography (eLORETA) of the alpha band comparing pre-rest and post-rest of hand massage (**A**) and foot massage (**B**). White blobs indicate increased activity at post-rest for each massage. Images depicting the SnPMs from different perspectives were based on voxel-by-voxel *t*-values of the differences. Compared with pre-rest for each massage, we observed a significantly increased alpha band in the left insular cortex at post-rest for hand massage (*p* < 0.05), and the left and right posterior cingulate cortex at post-rest for foot massage (*p* < 0.05). Structural anatomy is shown in gray scale (L, left; R, right; A, anterior; P, posterior).

Before the massage, there were no significant differences between the groups in terms of the age, height, and body weight of the participants (all *p* > 0.05, Table 1). The degree to which participants felt pleasant, relaxed, and refreshed was high for both hand and foot massage, but there was no significant difference observed between the effects for both body parts (*p* > 0.05, Table 2). Resting-state alpha activity significantly increased in the left insular cortex after hand massage compared with before massage (*p* < 0.05). Moreover, resting-state alpha activity significantly increased in the right and left posterior cingulate cortex after foot massage compared with before massage (*p* < 0.05) (Figure 1, Table 3).

## 4. Discussion

In this study, participants reported that they felt pleasant, relaxed, and refreshed to a high degree after both hand and foot massage, but no significant difference was observed between the massage types. According to our previous study [13], healthy women reported a point score of 4.5 ± 0.6 for pleasant, 4.1 ± 0.9 for relaxed, and 4.1 ± 0.8 for refreshed feelings after massage. We observed similar ratings of emotions and mood states after massage in elderly people requiring long-term care in this study. These results suggest that hand and foot massage as conducted in this study could act as a tool to improve the emotions and mood of elderly people requiring long-term care.

Moreover, we observed a significant increase in resting-state alpha activity in the left insular cortex after hand massage, and in the right and left posterior cingulate cortex after foot massage. Previous studies have demonstrated that left insular cortex activation is related to positive emotions [36,37]. In addition, it has been reported that activity in the insular cortex increases when participants are explicitly evaluating their feelings and representing them in awareness [38]. In contrast, research suggests that activity of the posterior cingulate cortex is related to recollection and memory of positive emotions [39,40]. In this study, because there was a high degree of pleasant, relaxing, and refreshing feelings after both hand and foot massage, we suggest that massage enhanced positive emotions by increasing resting-state alpha activity in the left insular cortex and the left and right posterior cingulate cortex. Moreover, it is possible that different brain regions were activated by hand and foot massage, which reflects different mechanisms underlying emotion changes related to massage of different body regions. Different brain regions were activated because the sensations felt in the body are different with the hand and foot massage [39].

The present study does have some limitations. First, because massage was conducted by two therapists, each of whom performed one type of massage, it is not clear whether the differences between the groups reflects the effect of the therapist or of the intervention. It is therefore necessary to verify whether similar results would be obtained with different types of massage being performed by the same therapist. Second, the long-term effects of massage and its effects on participants with any disease remain unclear. Future studies are needed to verify the effects of massage taking into consideration the above issues. Third, the sample size of this study was small and the results require confirmation in a larger group of patients in subsequent studies. Fourth, it was not clarified whether psychological factors changed from pre- to post-massage because the Likert scale was measured only after massage. It is necessary to measure psychological factors both before and after massage in future studies. In addition, a visual analogue scale including therapeutically relevant items, such as anxiety, depressive mood, and chronic pain should be included in the psychological evaluation. Fifth, the causal relationship between psychological factors and EEG activity was not clarified in this study. To investigate this causal relationship, studies should investigate the influence of reinforcement or suppression of EEG activity on the results by using non-invasive brain stimulation. Sixth, the emotional measure was not validated in this study. Future studies should measure detailed changes in emotions with validated scales to investigate the effectiveness of massage. Seventh, it remains unclear why different brain regions are activated by hand and foot massage. Future studies should analyze emotional changes after hand and foot massage in detail because positive and negative emotions are experienced in different body parts [41]. Eighth, there may be a potential bias in the emotional measure data because study investigators measured the post-massage data. It is necessary to verify the effect of massage using a blinded study design in future.

## 5. Conclusions

This study investigated the effect of hand and foot massage therapy on psychological factors and EEG activity in elderly people requiring long-term care. Results showed that the degree to which participants felt pleasant, relaxed, and refreshed was high after both kinds of massage. Moreover, resting-state alpha activity significantly increased in the left insular cortex after hand massage, and in the right and left posterior cingulate cortex after foot massage. This study suggests that hand and foot massage modulate psychological factors and EEG activity in elderly people requiring long-term care and can be used more regularly to support the well-being of elderly people.

## Figures and Tables

**Table 1:** Characteristics of group A (*n* = 6) and B (*n* = 6).

	Group A			Group B			*p*-Value
	Mean		SD	Mean		SD	
Age (years)	80.33	±	3.98	83.00	±	2.97	0.22
Height (cm)	152.67	±	8.10	147.77	±	5.99	0.26
Body weight (kg)	53.35	±	12.53	56.88	±	4.70	0.53
MMSE (scores)	27.67	±	2.42	26.50	±	2.43	0.42

MMSE: Mini-Mental State Examination.

**Table 2:** Comparison of emotions and mood states after hand and foot massage (*n* = 12).

	Hand Massage			Foot Massage			*p*-Value
	Mean		SD	Mean		SD	
Pleasant (scores)	4.17	±	0.72	4.08	±	0.67	0.78
Relaxed (scores)	3.92	±	1.00	4.17	±	0.83	0.56
Fresh (scores)	4.00	±	0.74	3.92	±	0.79	0.71

**Table 3:** Brain regions showing significantly higher activation in the alpha band at post-rest than at pre-rest for hand and foot massage (*n* = 12).

Type of Massage	Brain Region	BA	MNI Coordinates	*p*-Value
			(x, y, z)	
Hand	Insular cortex	13	35, 0, 15	<0.05
Foot	Left posterior cingulate cortex	31	10, −70, 15	<0.05
	Right posterior cingulate cortex	31	−10, −75, 20	<0.05

BA: Brodmann area; MNI: Montreal Neurological Institute.
